# A new classification scheme for laryngomalacia

**DOI:** 10.1007/s00405-025-09434-5

**Published:** 2025-05-13

**Authors:** H. Attya

**Affiliations:** Cairo University, Faculty of Medicine, ENT Department, Cairo University Children’s Hospital, Al Inshaa WA Al Munirah, El Sayeda Zeinab, Cairo Governorate, 11617 Egypt

**Keywords:** Laryngomalacia, Classification, Severity

## Abstract

**Objectives:**

To propose a novel classification method for assessing the severity of laryngomalacia (LM).

**Materials and methods:**

During a three-year period, all paediatric patients diagnosed with laryngomalacia were included. A disease-specific questionnaire consisting of eleven questions with four possible responses was administered to gather patient information, and a scoring system was utilized to classify the severity of their condition based on medical history and examination findings. Patients were assigned to two groups: group 1 included those having LM with no or mild co morbidities, group 2 included patients with severe co morbidities. Group 1 was further subdivided into three subgroups based on symptom scores [ (1a) with a score of 1–11, (1b) with a score of 12–22, and (1c) with a score of 23–33].

**Results:**

The study enrolled a total of fifty participants. Five individuals with laryngomalacia and severe comorbidities were placed in group 2, while the remaining forty-five participants were assigned to group 1. Initial assessment revealed that thirty-five participants were categorized in subgroup 1c and 10 in subgroup 1b. Individuals in subgroup 1c underwent supraglottoplasty and demonstrated an average 25% reduction in symptom scores at 6 weeks post-surgery.

**Conclusion:**

A severity-based classification system is proposed, and a management strategy is presented for laryngomalacia.

**Supplementary Information:**

The online version contains supplementary material available at 10.1007/s00405-025-09434-5.

## Introduction

Laryngomalacia is the most common congenital anomaly of the larynx. This is a dynamic abnormality that is characterized by supraglottic collapse. The degree of this collapse and the supraglottic obstruction that results, determines the severity of respiratory distress the patient may have [1].

Various classification systems exist in the literature but no uniform description of the underlying pathology or the surgery performed, known as supraglottoplasty [1–6]. The published literature has utilized the term "supraglottoplasty" to refer to a variety of surgical procedures targeting laryngomalacia, yet many studies have failed to include detailed descriptions of the specific surgical techniques that were used when employing this terminology [7]. A consensus statement stated that there is variation in practice among group members [8]. There is neither stratification nor correlation of clinical presentation, endoscopic appearance, treatment, and outcome [5].

Laryngomalacia is a condition that typically resolves on its own. However, in cases where children experience significant symptoms, surgery may be necessary. It is estimated that around 20% of affected patients require surgical intervention [1, 9–11]. There is variability in the literature regarding this percentage [12–14]. The criteria for determining the need for surgery are not well-defined. Symptoms such as apnea, recurrent cyanosis, failure to thrive, and persistent chest retractions suggest a more severe form of the disease [8].

There is an increasing demand for a credible and dependable system to determine the severity of laryngomalacia and assist in making informed decisions about the most suitable intervention. In this research, we have devised a proposal for evaluating patients with laryngomalacia. We share our experience as regards the management of this common condition.

## Materials and methods

### Study population

All children presented with clinical and endoscopic evidence of laryngomalacia during a three-year period were enrolled in this study. Evaluative data were collected in a combination of prospective and retrospective fashions from patients with congenital laryngomalacia evaluated by the author and colleagues at a large tertiary care paediatric referral centre. Patients with incomplete medical notes, associated synchronous airway lesions and older than 18 months were excluded.

### Assessment tools for the proposed classification

The assessment of patients with inspiratory stridor suggestive of laryngomalacia involves taking a detailed medical history and conducting an examination. To gather information about symptoms of airway obstruction, disease-specific patient questionnaire of eleven questions with four possible answers was applied and based on studies reporting LM symptoms [1, 3–6, 8–10]. The proposed classification implements a scoring system to categorize the presentation of patients based on their medical history, as described in Table 1.

**Table 1:** Disease-specific patient questionnaire

Question/Score	0	1	2	3
1. Does your child have noisy breathing	Rarely	Occasionally	Mostly/frequently	Persistently
2. In general, do you feel that your child is well fed?	Certainly	Probably	moderately	No
3. Does he choke and gasp while feeding?	Never	Rarely	Occasionally/sometimes	Mostly/frequently
4. Is there milk reflux?	Never	Rarely	Occasionally/some tomes	Mostly/frequently
5. How much milk does your baby receive for each feeding (age less than 2 months)?	Adequate 85–100 ml Feeds well	-Average 85–70 ml -Good/fair	−60–70 ml Average	- Less than 60 ml - Small/inadequate
5* How much milk does your baby receive for each feeding (age more than 2 months)?	Adequate e.g 110–130 ml	Good/fair e.g. 100–110	Average e.g90-100	Small/inadequate Less than 90
6. How long does it take to feed him (minutes)?	In a short time 10–15 min	15–25 min	25–35 min	More than 35 min Takes a long time to finish his feed
7. Does he stop feeding to catch his breath?	Never	Rarely	Occasionally/sometimes	Mostly/frequently
8. Breath holding or blue spells?	Never	Rarely	Occasionally/sometimes	Mostly/frequently
9. Recurrent chest infections	Never	Rarely	Occasionally/sometimes	Mostly/frequently
10. The ribs and neck are pulled inward when breathing/	Never	Rarely	Occasionally/sometimes	Mostly/frequently
11. “Does your child experience any of the following during sleep? Pauses in breathing, restless sleep. Or repeatedly waking up disturbed, coughing or choking	Never	Rarely	Occasionally/sometimes	Mostly/frequently

Furthermore, flexible nasal endoscopy (FNE) was conducted to assess vocal cord movement and detect signs of laryngomalacia. The results were recorded using a specific scoring system for laryngomalacia assessment based on the dynamic abnormalities observed during the awake FNE in addition to weight percentile per clinical growth charts as demonstrated in Table 2.

**Table 2:** Examination scores

Examination items/score	0	1	2
1. Supraglottic collapse	No	Moderate	Severe
2. Epiglottis	Normal	Omega	Tubular/severely folded
3. Arytenoid mucosa	No collapse	Moderate	Severe
4. Vocal cords visibility	Seen	Partially seen	Not seen
5. Epiglottis retroversion	No	Moderate	Severe
6. Weight percentile (not otherwise explained)	More than 25	25–5	Less than 5th

Both assessment tools; the questionnaire and the examination findings were given scores and categorised as in Table 3.

**Table 3:** Classification according to symptom and examination scores

Score/category	Mild	Moderate	Severe
Symptoms score	1–11	12–22	23–33
Examination score	1–4	5–8	9–12

### Classification

Participants were stratified into two groups based on symptom severity and comorbidities: group 1 comprised individuals with laryngomalacia and either no comorbidities or mild comorbidities such as medically managed reflux, while Group 2 consisted of children with severe/complex comorbidities or syndromes. Group 1 was further subdivided into three subgroups according to the severity of their condition, as indicated by their symptom scores. The eleven-question patient-reported symptom questionnaire featured a possible response score ranging from 0 to 3 for each question, resulting in a total score range of 0 to 33. Accordingly, patients were categorized into three subgroups, with each group assigned an equally spaced score range of 11 points. Given that higher scores indicate more severe symptoms, it was deemed appropriate to classify patients into three groups with ascending severity levels.

### Surgical intervention

**Fig. 1:**
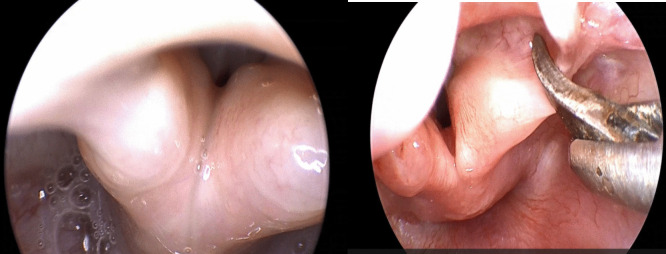
Division of AE folds; left: tight short AE folds causing supraglottic obstruction, Right: cold steel division of left AE fold

**Fig. 2:**
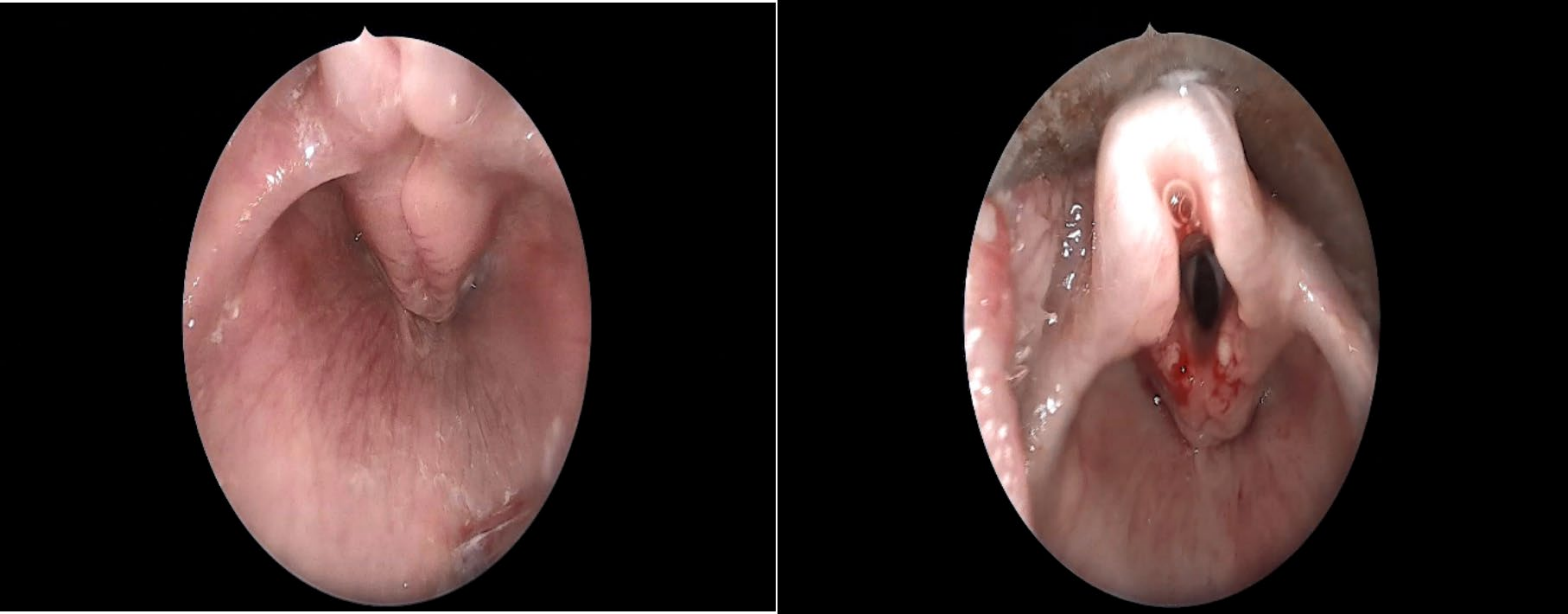
Excision of redundant arytenoid mucosa; left: introperative photo of the supraglottic collapse mainly by the arytenoid mucosa. Right: after excision of the aryteoid mucosa

**Fig. 3:**
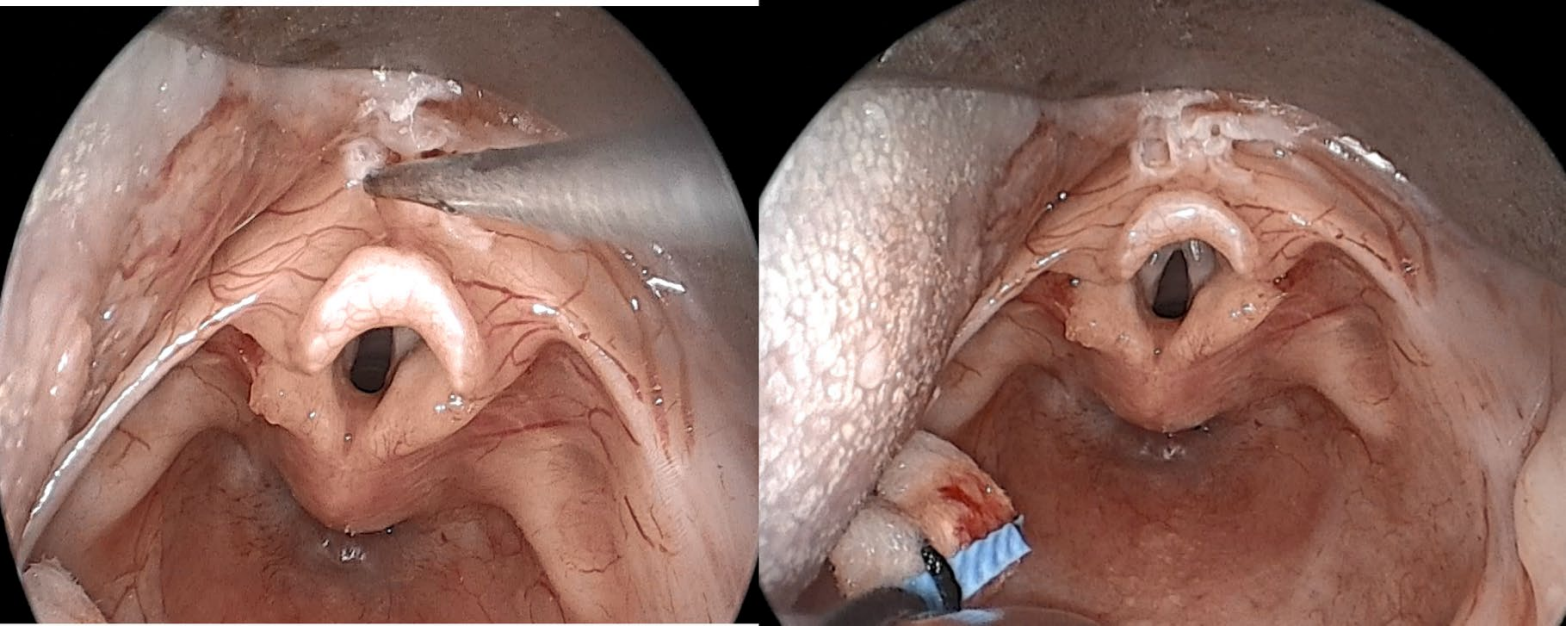
Epiglottopexy; left: gentle cauterization of the lingual surface of the epiglottis. Right: after cauterization; note the corrected retroversion of the epiglottis as the anterior commissure can be seen without changing the position of the laryngoscope

Supraglottoplasty was performed for all patients who were determined to have severe laryngomalacia and were categorized as belonging to subgroup 1c, which is characterized by a range of symptom scores from 22 to 33. The technique was tailored for each patient according to the sites of supraglottic collapse as identified from flexible endoscopy and intraoperative observation. The procedure involves one or more of the following surgical steps: division of AE folds (Fig. 1), excision of the redundant mucosa over arytenoid (Fig. 2), Epiglottopexy (Fig. 3).

### Conservative treatment

For mild cases (group 1a), patients are given an open appointment, and a copy of the questionnaire is given to the patient and instructed to repeat the questionnaire every two weeks and to send a copy to our dedicated email address.

For moderate cases (group 1b), assessment of GERD and treatment is started. A follow up appointment is scheduled. Should the child progress to the severe category based on the above detailed classification system, the decision is made to proceed with supraglottoplasty.

### Follow up

All cases whether managed conservatively or surgically were followed up at 6 weeks and 12 weeks with the questionnaire for symptoms score. Data from our medical records regarding those patients were reviewed and analysed.

### Statistical analysis

Data was entered and statistically analysed on the Statistical Package of Social Science Software program, version 25 (IBM SPSS Statistics for Windows, Version 25.0. Armonk, NY: IBM Corp.). Data was presented using mean and standard deviation for quantitative variables and frequency and percentage for qualitative ones. Comparison between groups for quantitative variables the comparison was conducted using Mann Whitney test (if 2 groups) or Kruskal Wallis test (if > 2 groups). Paired values were compared using paired t-test. Cronbach's Alpha a measure of the internal consistency of a test or scale; it is expressed as a number between 0 and 1 Correlation between different quantitative variables was performed using Spearman correlation coefficients. *P* values less than or equal to 0.05 were considered statistically significant. Score % change (improvement) calculated as follows.

$$6\text{weeks Score}\; \% \;\text{change }\left(\text{improvement}\right)=\frac{6\; \mathbf{w}\mathbf{e}\mathbf{e}\mathbf{k}\mathbf{s}\; \mathbf{s}\mathbf{c}\mathbf{o}\mathbf{r}\mathbf{e}-\mathbf{P}\mathbf{r}\mathbf{e}\mathbf{o}\mathbf{p}\mathbf{e}\mathbf{r}\mathbf{a}\mathbf{t}\mathbf{i}\mathbf{v}\mathbf{e}\; \mathbf{s}\mathbf{c}\mathbf{o}\mathbf{r}\mathbf{e} }{\mathbf{P}\mathbf{r}\mathbf{e}\mathbf{o}\mathbf{p}\mathbf{e}\mathbf{r}\mathbf{a}\mathbf{t}\mathbf{i}\mathbf{v}\mathbf{e}\; \mathbf{s}\mathbf{c}\mathbf{o}\mathbf{r}\mathbf{e}}\times 100$$

### Ethical considerations

The study was approved by the research ethics committee of our institution (N-456–2023). All data are anonymous, and it is impossible to identify patients from this report. I confirm that the procedures followed were in accordance with the ethical standards of the responsible committee on human experimentation and with the Helsinki Declaration of 1975, as revised in 2013.

## Results

In this study, a total of fifty patients were enrolled. The patients’ description as regards demographics, co morbidities, LM group assignments, preoperative and postoperative scores and treatment details are shown in online resource 1. Descriptive statistical analysis of data is presented and summarized in Table 4.

**Table 4:** Showing descriptive statistical summary of patients’ data

	Description [*n* = 50] (%)
**Age (month)**	
Range	1—18
Mean ± SD	5.1 ± 4.2
**Sex**	
Male	31(62)
Female	19(38)
**Co morbidities**	
Mild	8
Severe	5
No co morbidities	37
**Preoperative Category/group assignment**	
1b	10 (20)
1c	35 (70)
2	5 (10)
**Treatment modality**	
Surgical	40 (80)
Conservative	10 (20)
**Preoperative symptom score**	
Range	16—31
Mean ± SD	24.3 ± 3.5
**Preoperative examination score**	
Range	2—12
Mean ± SD	7.5 ± 2.4
**6 weeks symptom score**	
Range	13—29
Mean ± SD	18.6 ± 3.5
**12 weeks symptom score**	
Range	10—29
Mean ± SD	14.7 ± 4.6
**Procedure (further treatment)**	
Tracheostomy	4 (8)
SGP	2 (4)
None	44 (88)

**Fig. 4:**
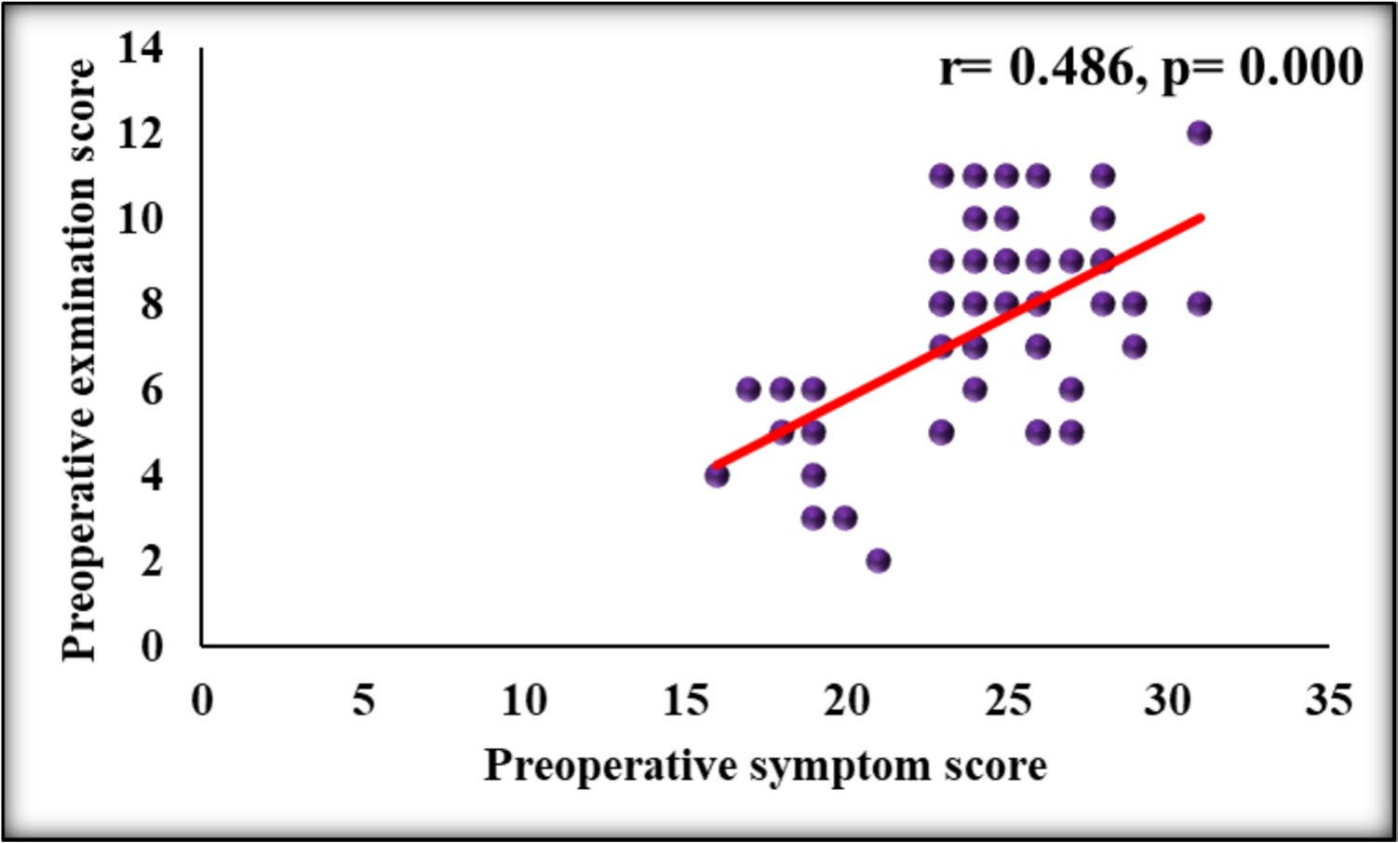
Scatter plot showing the correlation between Preoperative symptom score and preoperative examination score

The strength of the linear relationship between preoperative symptoms scores and preoperative examination scores was assessed by Spearman correlation coefficient. This revealed r value of 0.486 and *P* value of 0.000 (Fig. 4).

There were three subsites of supraglottic obstruction identified and their corresponding surgical corrections were conducted endoscopically. Those included: division of AF folds, excision of excess arytenoid mucosa and epiglottpexy.

Four cases in group 2 showed persistent symptoms after supraglottoplasty and needed tracheostomy. They had severe co morbidities such as severe swallow dysfunction, Arnold Chairi, Edward syndrome and severe cerebral palsy.

Table 5 shows comparison of 6 weeks score % change (improvement) regarding several factors. There was statistically significant improvement in symptom score when group 1c was compared to group 1b, and similarly when conservative treatment was compared to surgical treatment. Moreover, a significant difference was statistically demonstrated between preoperative score and 6 weeks scores (*P* value of 0.000) as shown in online resource 2.

**Table 5:** Comparison of 6 weeks score % change (improvement) regarding different factors

	6 weeks score % change (improvement)		*P* value
	Mean ± SD	Median (Range)	
**Sex**			
Male	‒23.8 ± 10.7	‒26.1 (−40.7—0)	0.582
Female	‒21.7 ± 14.2	‒20.8 (−48—3.9)	
**Category/group assignment**			
1b	‒12.3 ± 5.5	‒14.6 (−17.6—0)	1b VS 1c = 0.000
1c	‒28 ± 8.7	‒28 (−48—7.4)	1c VS 2 = 0.029
2	‒9.4 ± 17.9	‒3.7 (−40.7—3.9)	
**Treatment modality**			
S	‒25.7 ± 11.7	‒27.5 (−48—3.9)	0.000
C	‒12.3 ± 5.5	‒14.6 (−17.6—0)	

Overall Cronbach's Alpha value for questionnaire components is 0.715 which indicates an acceptable degree of internal consistency (reliability, harmony, concordance) of questionnaire components as shown in online resource 3.

## Discussion

Barthez and Rilliet [16], in 1843 described an infant with congenital stridor that improved by the age of 10 months. This is the first report of this condition which later has been known as laryngomalacia. Stridor is the most striking feature of this common condition and usually increase by crying, activity, supine position, and head flexion [17, 18].

**Fig. 5:**
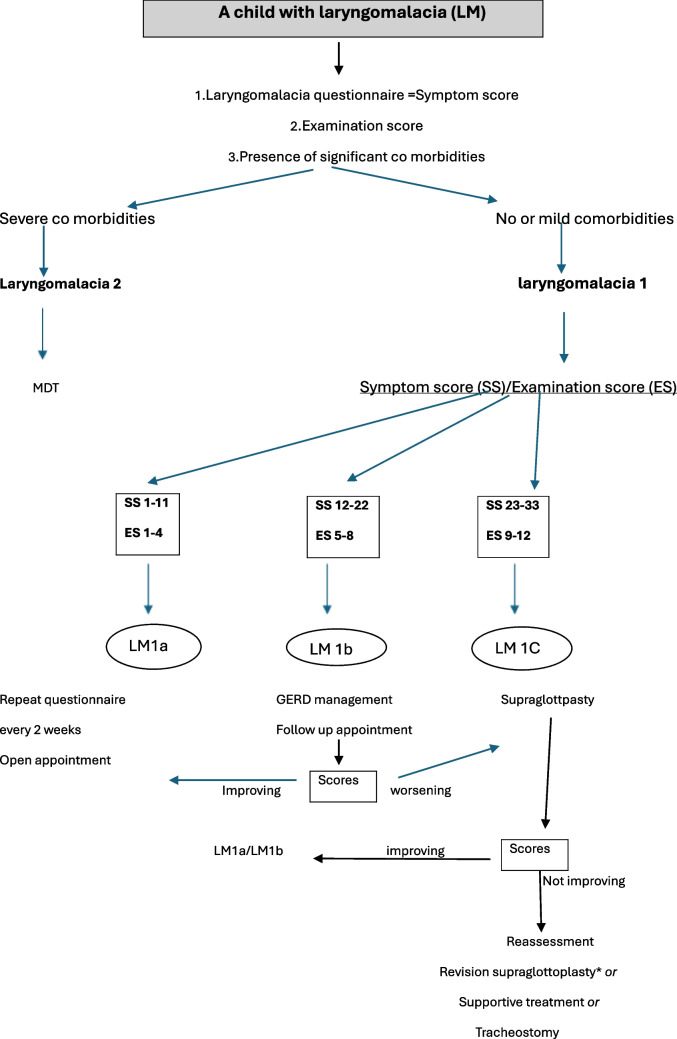
Management algorithm based on suggested laryngomalacia classification system. MDT =Multidisciplinary teams, *indicated particularly if persistent high examination scores from awake flexible endoscopy assessment. Supportive treatment may include feeding modification and advice, supplementary oxygen, GERD treatment

In this study, we propose a classification system based on the severity of symptoms, examination findings, and comorbidities. The system integrates these three factors to assist in clinical assessment, decision-making and surgical plan which can also enhance follow-up and the correlation between symptoms, signs, treatment modality and outcomes. Figure 5 demonstrates the suggested algorithm based on the proposed classification system.

One of the commonest classification systems for LM is that suggested by Olney in 1991 [1] however the classification does not address the frequent observations of overlapping sites of obstruction. Another system introduced by Hollinger [6] has less inter-rater reliability scores than Onley [15]

Thompson divided patients into three clinical groups according to disease severity based mainly on symptoms but the differentiation between moderate and severe cases was not clear. Furthermore, the suggested theory of aetiology does not adequately explain the fact that patients improve after suptaglottoplasty. The author observed no universal correlation between symptoms and signs of laryngomalacia in the article that presents a new theory of aetiology [9]

Obstructive sleep apnea (OSA) is highly prevalent among children with laryngomalacia. Retrospective studies have reported that up to 79% of children with LM also experience OSA [19]. Therefore, OSA evaluation was incorporated into the proposed patient questionnaire to quantify the severity of the condition.

Recently, a severity-based grading system has been developed by Roman [20] based on seven elements and seems to be relevant for determining the severity of laryngomalacia. However, its application becomes challenging when measuring oxygen saturation on various occasions and following up patients.

In our view, determining the severity of an airway problem such as laryngomalacia requires an accurate and thorough patient assessment. Relying on one consultation or a single snapshot of a child’s everyday breathing and feeding patterns may not be sufficient, which is why a questionnaire completed by the caregiver is believed to be more informative.

The article published by IPOG acknowledged the fact the variation does exist in the practice of its members. They suggested a comprehensive care algorithm that stratify laryngomalacia according to the type of symptoms. However, there is some overlap between moderate and severe LM in terms of feeding and breathing symptoms without a clear distinction [8].

The author's opinion is that the presence of severe symptoms and significant signs, indicated by high scores, suggests a good indication and a potentially positive outcome for supraglottoplasty. Conversely, severe symptoms with minimal signs may indicate more of a functional rather than structural pathology that cannot be corrected efficiently with supraglottoplasty. This pattern is commonly observed in cases with associated co-morbidities, resulting in a poorer response to supraglottoplasty and a higher likelihood of requiring tracheostomy.

The epiglottis behaviour as seen during FNE is a key component of the examination tool for the proposed classification. Grade 1 prolapse as described by Yellon RF [21], in which there is prolapse of the epiglottis against the posterior pharyngeal wall with obstruction of the airway, but normal position of the base of tongue, is of what is concerning here, and its correction has been previously described [22]. We believe that addressing epiglottis retroversion by epiglottopexy is important to achieve good outcome. Higher grade epiglottic and base‐of‐tongue prolapse entails tongue base contribution rather than epiglottis retroversion and hence no epiglottic surgery would be significantly beneficial.

Thompson stated that infants with additional health conditions are more prone to requiring repeated supraglottoplasty and tracheostomy [10]. The author suggests that these cases should be considered a distinct category, warranting an integrated management approach by multidisciplinary teams due to their complexity and severe symptoms that can be multifactorial. Therefore, our proposed system involves classifying these patients as a separate group.

Recent studies have explored the use of computer stress models to evaluate the severity of laryngomalacia. These studies suggest that while the von Mises stress peak can indicate cartilage abnormalities in the larynx, it may not accurately assess soft tissue abnormalities [23]. Therefore, caution should be exercised when using computer stress models for this purpose as their sensitivity may be low [24].

Swallowing issues in children with LM are thoroughly documented. The connection between reflux and the severity of LM is a topic of debate [25–28]. However, there is a general agreement to initiate acid suppression in cases of moderate and severe conditions [8]. Symptoms indicating reflux have been included in this classification proposal as an indication of disease severity due to observations confirming the interplay between reflux severity and its management with laryngomalacia and improvement in airway obstruction [25, 29–32].

The reliability of this proposal in patient selection for surgery is enhanced by the combination of symptom check and endoscopic findings and the statistically confirmed correlation between symptoms score and examination score. Moreover, in terms of postoperative follow-up, the questionnaire can assess patient reported outcomes, determine if further intervention is needed and detect case progression to identify those whose symptoms got worse and need surgical intervention.

While the proposed classification system appears to rely more on subjective symptom assessments, this approach is justified given the self-limiting and variable nature of laryngomalacia. The decision to intervene clinically is primarily driven by the severity of the patient's reported symptoms. Therefore, it is reasonable that the classification system should be correlated with treatment options and disease progression by focusing on symptomatology. Moreover, the inclusion of physical examination findings, which have been demonstrated to correlate with symptom scores, further strengthens the validity of this patient-centred classification methodology. Accordingly, parents are engaged in the assessment process and decision-making, enabling them to better understand the progression or improvement of the disease by completing a questionnaire and comprehending the category of their child's condition.

This study has potential limitations. The majority of cases enrolled in this study were those referred for ENT assessment at a tertiary paediatric hospital. Thus, many of them had significant symptoms. The retrospective data collection is another pitfall. Hence, further research is needed to address these issues and validate this scheme.

This system simplifies decision making for otolaryngologists to decide which patient need surgery and for paediatricians to consider referral to a paediatric ENT service, thus serving as a referral criterion for paediatric otolaryngology services. The framework is designed to be modifiable as knowledge evolves.

## Conclusions

There is a growing need for a reliable classification system for laryngomalacia that correlates with the appropriate surgical management. A novel classification system based on symptoms severity is presented and its correlation with patient reported outcome is studied. Thus, it paves the way for developing a management strategy for such a common paediatric airway disease.

## Supplementary Information

Below is the link to the electronic supplementary material.

Supplementary file1 (DOCX 33 KB)

Supplementary file2 (DOCX 22 KB)

Supplementary file3 (DOCX 23 KB)

## Data Availability

The datasets used in and/or analysed in the current study are available from the corresponding author upon a reasonable request.
