# Supplementary material for: A new classification scheme for laryngomalacia
Source: Eur Arch Otorhinolaryngol. 2025 May 13;282(7):3637–46. doi: 10.1007/s00405-025-09434-5 (PMC12321643; doi:10.1007/s00405-025-09434-5)
Supplement: Supplementary file 1 — Supplementary file1 (DOCX 33 KB) [file 405_2025_9434_MOESM1_ESM.docx]

| N | Age  months | sex | Comorbidities | Preoperative symptom score/examination score | Group assignment / Treatment modality | 6 weeks follow up score post treatment (conservative or surgical) | 12 weeks follow up score post treatment  /New group assignment | Further  treatment |
| --- | --- | --- | --- | --- | --- | --- | --- | --- |
| 1 | 2 | M | NA | 16/4 | 1b/C | 15 | 11/ 1a | NA |
| 2 | 5 | M | NA | 19/3 | 1b/C | 16 | 12/1b | NA |
| 3 | 10 | M | NA | 24/6 | 1c/S | 18 | 14/1b | NA |
| 4 | 5 | F | NA | 25/10 | 1c/S | 13 | 11/1a | NA |
| 5 | 7 | F | NA | 24/10 | 1c/S | 19 | 13/1b | NA |
| 6 | 2 | F | NA | 21/2 | 1b/C | 18 | 14/1b | NA |
| 7 | 18 | M | NA | 26/5 | 1c/S | 20 | 16/1b | NA |
| 8 | 2 | M | NA | 23/9 | 1c/S | 18 | 15/1b | NA |
| 9 | 2.5 | M | NA | 25/11 | 1c/S | 18 | 15/1b | NA |
| 10 | 6 | M | NA | 28/11 | 1c/S | 25 | 21/1b | Revision SGP |
| 11 | 1.5 | F | NA | 26/8 | 1c/S | 20 | 14/1b | NA |
| 12 | 15 | M | Severe Cerebral Palsy | 29/7 | 2/S | 29 | NA | Trachestomy |
| 13 | 1 | F | Severe swallowing dysfunction-severe reflux necessitating gastrostomy and fundoplication | 26/7 | 2/S | 27 | NA | Trachestomy |
| 14 | 4 | M | Edward | 27/9 | 2/S | 26 | NA | Trachestomy |
| 15 | 5 | F | Arnold Chiari | 31/8 | 2/S | 29 | NA | Trachestomy |
| 16 | 12 | M | NA | 25/9 | 1c/S | 17 | 14/1b | NA |
| 17 | 10 | M | NA | 25/9 | 1c/S | 18 | 13/1b | NA |
| 18 | 6 | F | NA | 26/9 | 1c/S | 19 | 15/1b | NA |
| 19 | 4 | M | NA | 28/9 | 1c/S | 18 | 13/1b | NA |
| 20 | 15 | M | NA | 17/6 | 1b/C | 14 | 12/1b | NA |
| 21 | 4 | M | NA | 26/7 | 1c/S | 19 | 14/1b | NA |
| 22 | 3 | M | Mild cerebral palsy | 29/8 | 1c/S | 19 | 15/1b | NA |
| 23 | 7 | M | Cardiac PFO | 26/5 | 1c/S | 18 | 11/1a | NA |
| 24 | 3 | M | Severe Cerebral palsy | 27/5 | 2/S | 16 | 12/1b | NA |
| 25 | 2 | M | NA | 23/7 | 1c/S | 19 | 14/1b | NA |
| 26 | 1 | F | NA | 25/8 | 1c/S | 20 | 16/1b | NA |
| 27 | 6 | M | NA | 19/5 | 1b/C | 17 | 15/1b | NA |
| 28 | 9 | F | NA | 25/9 | 1c/S | 15 | 10/1a | NA |
| 29 | 10 | M | NA | 18/5 | 1b/C | 16 | 14/1b | NA |
| 30 | 2 | F | Mild swallowing dysfunction | 23/5 | 1c/S | 20 | 19/1b | NA |
| 31 | 10 | F | NA | 19/6 | 1b/C | 19 | 23/1b | Supraglottoplasty |
| 32 | 6 | M | NA | 23/8 | 1c/S | 17 | 13/1b | NA |
| 33 | 7 | M | Mild swallowing dysfunction/neurological impairment/micrognathia | 23/11 | 1c/S | 19 | 12/1b | NA |
| 34 | 1 | F | Reflux controlled with antireflux | 27/6 | 1c/S | 25 | 14/1b | NA |
| 35 | 1 | M | NA | 28/10 | 1c/S | 18 | 13/1b | NA |
| 36 | 2 | M | NA | 19/4 | 1b/C | 16 | 14/1b | NA |
| 37 | 5 | F | NA | 20/3 | 1b/C | 17 | 13/1b | NA |
| 38 | 13 | F | Mild Swallow dysfunction | 18/6 | 1b/C | 15 | 11/1a | NA |
| 39 | 3 | F | NA | 26/8 | 1c/S | 16 | 14/1b | NA |
| 40 | 1 | M | NA | 24/9 | 1c/S | 17 | 13/1b | NA |
| 41 | 1 | M | NA | 24/7 | 1c/S | 18 | 12/1b | NA |
| 42 | 2 | M | NA | 24/7 | 1c/S | 16 | 11/1a | NA |
| 43 | 4 | F | NA | 26/11 | 1c/S | 17 | 10/1a | NA |
| 44 | 1.5 | F | Multifenestrated ASD | 23/5 | 1c/S | 18 | 13/1b | NA |
| 45 | 1 | M | NA | 24/8 | 1c/S | 16 | 11/1a | NA |
| 46 | 5 | f | NA | 31/12 | 1c/S | 20 | 14/1b | NA |
| 47 | 4.5 | M | NA | 28/9 | 1c/S | 19 | 13/1b | NA |
| 48 | 3 | M | NA | 24/11 | 1c/S | 16 | 11/1a | NA |
| 49 | 2.5 | F | NA | 23/8 | 1c/S | 15 | 12/1b | NA |
| 50 | 2 | M | Reflux (controlled) | 28/8 | 1c/S | 18 | 12/1b | NA |

On line resource 1 the table demonstrates patients data
