# Supplementary material for: A new classification scheme for laryngomalacia
Source: Eur Arch Otorhinolaryngol. 2025 May 13;282(7):3637–46. doi: 10.1007/s00405-025-09434-5 (PMC12321643; doi:10.1007/s00405-025-09434-5)
Supplement: Supplementary file 2 — Supplementary file2 (DOCX 22 KB) [file 405_2025_9434_MOESM2_ESM.docx]

| **Score** | **MD** | **SD of MD** | **SE of MD** | **95% CI of MD** | | **t** | **P value** |
| --- | --- | --- | --- | --- | --- | --- | --- |
| **Preoperative symptoms score versus 6 weeks score** | 5.7 | 3.3 | 0.5 | 4.8 -6.7 | 12.1 | | **0.000** |

On line resource 2 The table demonstrates **Comparison of scores**
