# Supplementary material for: A new classification scheme for laryngomalacia
Source: Eur Arch Otorhinolaryngol. 2025 May 13;282(7):3637–46. doi: 10.1007/s00405-025-09434-5 (PMC12321643; doi:10.1007/s00405-025-09434-5)
Supplement: Supplementary file 3 — Supplementary file3 (DOCX 23 KB) [file 405_2025_9434_MOESM3_ESM.docx]

**A new classification scheme for laryngomalacia.**

**H attya 1 MD**

**ENT lecturer**

**1Cairo university, Faculty of medicine, ENT department, Cairo university children’s hospital**

**Corresponding author and for reprint request: Dr. Hisham M Anwar Attya**

**Email: hesham_anwar@cu.edu.eg. Drhishamattya1@gmail.com**

**European archives of otorhinolaryngology**

Cronbach's Alpha a measure of the internal consistency of a test or scale; it is expressed as a number between 0 and 1. Internal consistency describes the extent to which all the items in a test measure (questionnaire) the same concept or construct and hence it is connected to the inter-relatedness of the items within the test.


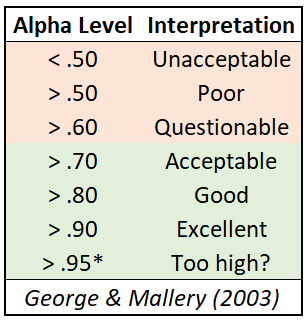


**Calculating Cronbach's Alpha for the questionnaire after deleting each question**

|  | **Cronbach's Alpha if Item Deleted** |
| --- | --- |
| **Q1** | 0.708 |
| **Q2** | 0.687 |
| **Q3** | 0.664 |
| **Q4** | 0.702 |
| **Q5** | 0.724 |
| **Q6** | 0.716 |
| **Q7** | 0.690 |
| **Q8** | 0.669 |
| **Q9** | 0.724 |
| **Q10** | 0.659 |
| **Q11** | 0.686 |
